# Supplementary material for: MetaRibo-Seq measures translation in microbiomes
Source: Nat Commun. 2020 Jun 29;11:3268. doi: 10.1038/s41467-020-17081-z (PMC7324362; doi:10.1038/s41467-020-17081-z)
Supplement: Supplementary file 10 — Supplementary Data 7 [file 41467_2020_17081_MOESM10_ESM.zip › File2/Confidence_VeryHigh_Taxonomy/291484_out.krona.html]

Javascript must be enabled to view this page.

members
magnitude
magnitudeUnassigned
count
unassigned
taxon
rank

291484\_out

7

7
superkingdom
2

phylum
7
1224

1236
7
class

91347
order
7

7
family
1

SRS019068\_contig\_number\_62048
543

570
genus
2
3

SRS050422\_contig\_number\_contig-100\_29978.101582SRS143070\_contig\_number\_10151

1
species

SRS077646\_contig\_number\_contig-100\_252.253
573

547
3
genus

species
1
539813

SRS1055050\_contig\_number\_contig-100\_45.10437

1
species group
2

SRS143070\_contig\_number\_contig-100\_2894.92799
354276

1
species

SRS012849\_contig\_number\_15845
158836
